# Soil and brownfield bioremediation

**DOI:** 10.1111/1751-7915.12840

**Published:** 2017-08-22

**Authors:** Mallavarapu Megharaj, Ravi Naidu

**Affiliations:** ^1^ Global Centre for Environmental Remediation and Cooperative Research Centre for Contamination Assessment and Remediation of the Environment Faculty of Science University of Newcastle Callaghan NSW 2308 Australia

## Abstract

Soil contamination with petroleum hydrocarbons, persistent organic pollutants, halogenated organic chemicals and toxic metal(loid)s is a serious global problem affecting the human and ecological health. Over the past half‐century, the technological and industrial advancements have led to the creation of a large number of brownfields, most of these located in the centre of dense cities all over the world. Restoring these sites and regeneration of urban areas in a sustainable way for beneficial uses is a key priority for all industrialized nations. Bioremediation is considered a safe economical, efficient and sustainable technology for restoring the contaminated sites. This brief review presents an overview of bioremediation technologies in the context of sustainability, their applications and limitations in the reclamation of contaminated sites with an emphasis on brownfields. Also, the use of integrated approaches using the combination of chemical oxidation and bioremediation for persistent organic pollutants is discussed.

## Introduction

Anthropogenic activities such as industrial, mining and military processes are the major sources that contributed to widespread contamination of the environment throughout the world with numerous chemicals including petroleum hydrocarbons, polyaromatic hydrocarbons (PAHs), polychlorinated biphenyls (PCBs), halogenated dibenzodioxins/furans, chlorinated solvents, pesticides and toxic heavy metal(loid)s. Consequently, several thousands of sites around the world are seriously polluted requiring remediation. The costs for cleaning up of contaminated sites are extremely high, and in the USA alone about $6–8 billion is spent annually. Global costs are in the range of 425–500 billion (Glass, 1999; Tsao, 2003). Traditional methods for remediation of contaminated soils include dig and dump, excavation, transport, landfilling, soil washing, the addition of oxidants (hydrogen peroxide or potassium permanganate) and incineration. Due to the high cost of remediation technologies, several polluted commercial properties were abandoned or idled rather than remediated. There are over 500 000 of these so‐called brownfields in the USA (Doty, 2008) with an estimated clean‐up and redevelopment costs more than $650 million (Bressler and Hannah, 2000). Almost 800 000 potential brownfield sites have been identified in Europe (Oliver *et al*., 2005). According to European official reports (EEA, 2000; Van‐Camp *et al*., 2004), the total clean‐up costs for the countries that have provided data were estimated as about 115 billion euros, or 490 euros/capita. According to the German register of contaminated sites, there are about 300 000 potentially contaminated sites (UBA, 2015).

The land is scarce which supports life on earth and soil is not a renewable resource. Cleaning up of contaminated soil and its protection are key priorities for redeveloping land and urban regeneration in developed or industrialized countries. Industrialization together with technological advancements over the past more than 60 years has led to the creation of large areas of abandoned or underused and potentially contaminated lands in cities and suburbs throughout the world, and these are classified as brownfield sites. As the cities grew outwards, brownfields became located in the centre of cities often occupying high‐value lands. Brownfield sites pose a risk to human and environmental health have negative impacts on the economy at the regional level by becoming obstacles for urban development; therefore, cleaning up of these sites have become priorities for many nations. According to the USEPA (2002) brownfield site is “real property, the expansion, redevelopment or reuse of which may be complicated by the presence or potential presence of a hazardous substance, pollutant or contaminant”. Brownfields contain co‐contaminants. For the past decade, there has been an increasing awareness and interest among the public for sustainability in remediation, especially in the developed countries. Sustainable remediation not only brings great opportunities but also challenges, for both researchers and the practitioners in the remediation area. Sustainability considers that the resources are finite and should be used judiciously to meet the needs of current but without compromising the future generations. Thus, the benefits of sustainable remediation are realized through the promotion of renewable energy, material recycling, preservation of natural resources and minimization of waste and energy. The traditional physicochemical technologies for soil remediation cannot be considered as sustainable because these technologies do not include the criteria for sustainability mentioned above. Over the past decade, green and sustainable remediation is gaining importance as a beneficial approach to optimize all phases of remediation. Bioremediation mediated by biological agents such as microorganisms (bacteria, fungi, algae, etc.) or plants is considered a cost effective, green and sustainable approach for restoring the contaminated sites. However, bioremediation has its limitations for its field‐scale application as an efficient remediation technology other than for petroleum hydrocarbon contaminated sites. The available remediation technologies including bioremediation for both organic and inorganic contaminants have been critically reviewed (Megharaj *et al*., 2011; Chen *et al*., 2015; Kuppusamy *et al*., 2016a,b). This article presents an overview of bioremediation technologies in the context of sustainability, their applicability and limitations for reclamation of contaminated sites with an emphasis on brownfield sites. Also, the advantages of integrated bioremediation technologies in combination with other technologies where bioremediation alone is not efficient is discussed with some examples.

## Bioremediation approaches

Bioremediation approaches can be applied either *in situ* or *ex situ* depending on the nature of contaminant and site conditions. *In situ* treatment is more attractive and cost effective as it is not or less disruptive and does not involve excavation and transport of contaminated soils. The commonly used in situ approaches include natural attenuation, biostimulation, bioventing and bioaugmentation. In contrast, the *ex situ* approaches involve excavation and removal of contaminated soil for treatment either on the site or transportation to a suitable place before treatment. The commonly used *ex situ* bioremediation approaches include land farming, biopiles and bioslurries. Each contaminated site or brownfield represents a challenge due to its former use and depending on whether it is abandoned or underused, and the contamination is real or perceived. Biotechnological interventions are required to bring back these sites to their beneficial uses. Bioremediation approaches when combined with sustainable practices such as the use of renewable sources (e.g., solar or wind power instead of fossil fuel based energy or generation of biomass for bioenergy) will result in greater environmental, economical and societal benefits.

## Natural attenuation

Natural attenuation processes involve contaminant attenuation to harmless products through natural processes, such as microbial degradation, volatilization, sorption and immobilization. The natural attenuation process is contaminant specific and commonly employed for petroleum hydrocarbon contaminated sites. However, natural attenuation may not be a suitable option for several other contaminants such as persistent organic pollutants. Although natural attenuation has proven to be a successful approach to treat petroleum contaminants (benzene, toluene, ethylbenzene and xylene), it may not work if the site does not have the contaminant degrading microorganisms or nutrients.

## Biostimulation

The microbial transformation of contaminants in soils depends on the availability of nutrients (carbon, nitrogen, phosphorus and potassium), favourable environmental conditions (pH, electrical conductivity, aeration, temperature) and the nature of contaminant itself and its bioavailability. Some contaminants such as persistent organic pollutants (e.g., PAHs, PCBs, lindane, dichlorodiphynyltrichloroethane) are extremely insoluble in water and tend to strongly sorb to organic matter in soils thereby decreasing their availability to microbes. The use of biosurfactants can enhance the bioavailability of such pollutants. The addition of slow release fertilizers or organic waste and manures can supply the nutrients and stimulate the indigenous microbes to transform the contaminants.

The addition of natural organic substrates such as mulch and manure has shown to remove perchlorate through stimulation of anaerobic degradation by microbes (USEPA, 2005). Perchlorate reducing bacteria are ubiquitous, have the ability to reduce perchlorate to chloride under anaerobic conditions using perchlorate as a terminal electron acceptor for growth and energy in the presence of electron donor (Waller *et al*., 2004). The bioremediation process using glycerine‐diammonium phosphate (DAP) successfully treated over 160,000 tonnes of soil from a 1000 acre Bermite site from Los Angeles, California containing 0.59–8.4 mg perchlorate/kg soil to non‐detectable levels within seven month period, which is considered to be a safe and economical treatment ($35 per tonne). The former Bermite site was used to manufacture various explosives and related products including perchlorate during 1934–1987 (Evans *et al*., 2008).

## Composting

The addition of compost or composting is considered to be one of the most cost‐effective approaches to remediate contaminated soils because it can increase soil organic matter content and soil fertility besides enhancing bioremediation. Several studies have demonstrated the effectiveness of composting as a technology to detoxify or stabilize a wide range of contaminants including toxic metals, PAHs and pesticides (Semple *et al*., 2001; Tandy *et al*., 2009; Zeng *et al*., 2011). Sorption of organic contaminants to soil organic matter can decrease the fraction of contaminant, that is available to microorganisms for degradation. However, water extractable organic matter from cow manure compost was shown to increase the solubility of certain PAHs phenanthrene, pyrene and benzo‐a‐pyrene with 8.4, 34 and 89 times higher than their measured concentrations in water, respectively, which enhanced their biodegradation (Kobayashi *et al*., 2009). The observed increase in PAH solubility and biodegradation was attributed to the high molecular weight (>1000 Da) fraction of water extractable organic matter from cow manure. In another study, Wu *et al*. (2013) demonstrated the enhanced bioavailability and removal of PAHs up to 90% in soils contaminated with diesel, coal tar and coal ash when amended with compost. Both degradation and desorption processes were attributed as reasons for the observed PAH disappearance. Degradation of organic contaminants in soil is often difficult due to their low bioavailability. The addition of surfactants to soil can increase the bioavailability of some organic pollutants (Cheng *et al*., 2008). Co‐composting of PAH polluted sediments with green waste in different proportions for nine months has resulted in a decrease of PAH concentrations to < 1 mg g^−1^ (Mattei *et al*., 2016). The co‐composted product is considered to have the potential for use as technosol or plant growth substrate in revegetation of urban areas or brownfields.

Pelaez *et al*. (2013) has successfully demonstrated field‐scale bioremediation of 900 m^3^ PAH polluted soil from a former chemical factory near Oviedo (Spain) used for manufacture of naphthalene, phenols and other chemicals from coal processing, in a biopile using commercially available fertilizer and surfactants, which resulted in 94.4% decrease in PAH contamination during 161 days. The decrease in PAHs coincided with an increase in indigenous bacteria able to degrade PAHs, with *Bacillus* and *Pseudomonas* being abundant bacteria.

## Bioaugmentation

Introducing specific microorganisms to decontaminate the soils when indigenous microbes are not efficient is considered a more acceptable approach to remediate the contaminated soils. However, the strains for bioaugmentation should ideally have (i) superior ability to degrade the target contaminants, (ii) easy to cultivate, (iii) fast growth, (iv) tolerance to the high concentration of contaminant and (v) ability to survive in a wide range of environmental conditions/stressors. Bioaugmentation has been proven to be successful for a wide range of pollutants including pesticides such DDT, lindane, endosulfan, pentachlorophenol (PCP), polyaromatic hydrocarbons (PAHs) and total petroleum hydrocarbons (Abhilash *et al*., 2011; Saez *et al*., 2014; Wang *et al*., 2014; Chen *et al*., 2015; Kuppusamy *et al*., 2016a,b). However, predation, competition and toxins in soils can negatively affect the survival of introduced microbes. In such cases, bioaugmentation using immobilized cells in carrier materials or preadapted strains to the problem soil conditions may prove to be advantageous regarding enhancing their survival in soils.

## Phytoremediation

The use of plants to remediate contaminated sites has been considered as an *in situ* cost‐effective option alternative to the relatively expensive traditional physicochemical technologies based on excavation, dig and dump. However, phytoremediation did not find wide application, especially for metal contaminated sites, due to potential risks to biota via the metal laden biomass. Phytostabilization rather than phytoaccumulation could be an attractive alternative option for remediation of metal contaminated sites. Phytostabilization involves stabilization/immobilization of contaminants in the soil via binding to the roots or complexation through root exudates, which reduces the bioavailability of contaminants, therefore, reduces the risk to food chain. Two heavy metal (Cu, Pb, Zn) contaminated brownfield sites (a former landfill site and an industrial site used for shipyard, wood impregnation, etc.) have been successfully remediated using phytostabilization through willow plants (*Salix Klara* and *Salix singer*). This field trial has demonstrated that phytostabilization of brownfield sites with bioenergy crops can provide environmental benefits by turning these areas into economical and beneficial uses (Enell *et al*., 2016). Plants in association with microbes can be applied to remove the labile/bioavailable pool of inorganic contaminants from a site, remove or degrade organic contaminants, stabilize or immobilize contaminants (phytostabilization/in situ immobilization/Phyto‐exclusion). (Vangronsveld *et al*., 2009; Mench *et al*., 2010).

Aided phytostabilization was applied over a six‐year period on a 1 ha site previously used for on‐land disposal of Zn, Pb and Cd contaminated sediments at Fresnes‐Sur‐Escaut in northern France. A basic mineral amendment (Optiscor™) was applied to the soil, which was then planted at high density with a commercial cultivar of grass (*Deschampsia cespitose*) (Bert *et al*., 2009, 2012). The trial showed stabilization of contaminants with effectively 100% vegetation cover (by reducing soil‐human contact via direct soil exposure and dust inhalation) and a reduction in plant‐metal uptake and transfer. Metal concentrations in the foliage of cover grass were reduced by 60% for Zn and 20% for Cd. Metal concentrations in biomass were sufficiently low to allow subsequent biomass use as compost. In Austria, *in situ* immobilization/Phyto‐exclusion was applied over a 13‐year period at Arnoldstein (South Austria) on arable land impacted by Pb/Zn smelter emissions. Gravel sludge and iron bearing materials (red mud) were applied as soil amendments and Cd excluding cultivars of commercial food crops (barley, maize and potatoes) grown with the aim of reducing contaminant transfer from soil to plants and groundwater (Friesl‐Hanl *et al*., 2009). Amendment addition resulted in a significant reduction in the labile contaminant pool (80% Cd; Zn > 90% and Pb > 90%) in the soils. Whereas, the Cd uptake by barley was decreased by > 75% compared to an accumulating cultivar. Uptake of Zn, Cd and Pb into maize silage was reduced by 70%, 60% and 50% respectively. Application of soil amendments (such as lime, red mud, zeolites, cyclonic ashes, iron grits and slags, composts, biochar and other organic amendments) has shown to reduce the bioavailability of a wide range of contaminants while simultaneously contributing to revegetation success and thereby, protecting against offsite movement of contaminants by wind and water (Bes and Mench, 2008; Vangronsveld *et al*., 2009; Jones *et al*., 2016).

Thus, phytoremediation has emerged as a promising strategy for *in situ* removal of a wide variety of contaminants (Gerhardt *et al*., 2009). Plants in association with microbes seem to be more effective for removal/degradation of organic contaminants from impacted soils.

About 40% of plant photosynthates are released as sugars, organic acids and other larger organic compounds into soils, which serve as carbon and energy sources for microbes (Leigh *et al*., 2002; Kumar *et al*., 2006). The flavonoids and coumarins that are released by plant roots can stimulate the growth and activity of PAH and PCB degrading bacteria (Leigh *et al*., 2002). During a 60‐week study, about a 73% decrease in total PAHs was observed in planted sediments compared with unplanted sediments which showed only 25% decrease (Huesemann *et al*., 2009). Phytoremediation over a two‐year period decreased the total PAH concentration by 30% which is double the unvegetated highly contaminated site (Siciliano *et al*., 2003). In a 60‐day field trial, 96% of 2,4,6‐trinitrotoulene was removed from a test plot by maize (*Zea mays*) (Dillewijn *et al*., 2007). The disadvantages of phytoremediation are that it is a slow process requiring several years and more crop harvests and the challenge is that there are stressors (variation in temperature, nutrients, precipitation, herbivory, plant pathogens, and competition by weeds) that affect phytoremediation in the field but are not encountered in the greenhouse. A successful strategy for overcoming the challenge of plant stress is to use plant growth promoting bacteria that can lower the level of deleterious ethylene and also enhance germination and plant growth rates under stress conditions, particularly when used in conjunction with contaminant tolerant plants species. Plant growth promoting rhizobacteria can also act as biocontrol agents by suppressing the plant pathogens.

## Integrated approaches

In most cases, single remediation technology may not be effective and requires a combination of technologies. Poor bioavailability of persistent organic pollutants (POPs) in soil often impedes the success of bioremediation as a feasible decontamination approach. Fenton—bioremediation is emerging as a promising integrated approach, which enhances POP removal efficiencies. Fenton oxidation followed by bioremediation could improve the effectiveness of bioremediation of highly contaminated soils. The integrated technology combines rapid and aggressive oxidation by Fenton pre‐treatment followed by degradation by microbial activity in the pre‐treated soil matrix. Efficiencies ranging from 70% to 98% have been reported for combined bioremediation‐Fenton treatment for POP contaminated soils (Gan and Ng, 2012). Fenton oxidation combined with bioremediation enhances PAH removal efficiency in several ways (Palmroth *et al*., 2006; Gan and Ng, 2012). Kao and Wu (2000) developed a combined Fenton pre‐treatment and bioremediation method to efficiently degrade 2,3,7,8‐tetrachloro dibenzo‐*p*‐dioxin (TCDD)‐contaminated soils. In this study, Fenton pre‐treatment removed 98% TCDD. The advantages of Fenton pre‐treatment are (i) decrease in pollutant concentrations to levels that are less toxic to soil biota, (ii) improvement of the bioavailability of parent PAH, (iii) prevention of incomplete mineralization of partially oxidized PAHs by utilizing degrading bacteria and fungi which are commonly found in the environment, (iv) release of oxygen from the H_2_O_2_ decomposition from Fenton treatment that provides aeration for aerobic biological transformation.

## Challenges and prospects

Large areas of land around the world have been impacted by former industrial and other anthropogenic activities. These include urban brownfields, former mining and resource extraction sites and bringing these back to beneficial uses require site‐specific approaches. Although bioremediation is considered environmentally beneficial and sustainable, the process can be slow. Current bioremediation technologies suffer from some limitations, which include the lack of adequate understanding of the contaminant degrading capabilities of microbial communities in the field, low bioavailability of contaminants on spatial and temporal scales and lack of adequate knowledge on metabolic cooperation networks among the microbial consortia/communities.

The restoration of natural functions of some contaminated sites may not be feasible and, hence, the application of the principle of function‐directed remediation may be sufficient to minimize the risks of pollutants and bring back the lands to beneficial uses. Integrated approaches such as pre‐treatment of highly contaminated soils using chemical oxidants in safe concentrations to soil biota, followed by bioremediation, appear to be a promising technology for some of the intractable pollutants. Also, plant‐microbe associations have great potential for their application in remediation of contaminated sites. Bioremediation, although green and environmentally safe, should be combined with renewable resources such as the wind, solar energy, and linked to the generation of biomass for renewable energy resources, all of which make bioremediation a more sustainable technology. Successful adaptation of sustainability in remediation is essential, and a concerted action of academia, government and industry are needed for successful implementation.

## Conflict of interest

The authors declare no conflict of interest.

## References

[mbt212840-bib-0001] Abhilash, P.C. , Srivastava, S. , and Singh, N. (2011) Comparative bioremediation potential of four rhizospheric microbial species against lindane. Chemosphere 82: 56–63. 21044795 10.1016/j.chemosphere.2010.10.009

[mbt212840-bib-0002] Bert, V. , Seuntjens, P. , Dejonghe, W. , Lacherez, S. , Thuy, H.T. , and Vandecasteele, B. (2009) Phytoremediation as a management option for contaminated sediments in tidal marshes, flood control areas and dredged sediment landfill sites. Environ Sci Pollut Res Int 16: 745–764. 19533193 10.1007/s11356-009-0205-6

[mbt212840-bib-0003] Bert, V. , Lors, C.H. , Ponge, J.F. , Caron, L. , Biaz, A. , Dazy, M. , and Masfaraud, J.F. (2012) Metal immobilisation and soil amendment efficiency at a contaminated sediment landfill site: a field study focusing on plants, springtails, and bacteria. Environ Pollut 169: 1–11. 22647548 10.1016/j.envpol.2012.04.021

[mbt212840-bib-0004] Bes, C. , and Mench, M. (2008) Remediation of copper contaminated top soils from a wood treatment facility using in situ stabilisation. Environ Pollut 156: 1128–1138. 18486289 10.1016/j.envpol.2008.04.006

[mbt212840-bib-0005] Bressler, A.J. , and Hannah, J.A. (2000) Brownfield redevelopment: a risk verses reward, 2000. URL Wwww.irmi.com/Expert/Articles/2000/Hannah12.aspx.

[mbt212840-bib-0006] Chen, M. , Xu, P. , Zeng, G. , Yang, C. , Huang, D. , and Zhang, J. (2015) Bioremediation of soils contaminated with polycyclic aromatic hydrocarbons, petroleum, pesticides, chlorophenols and heavy metals by composting: applications, microbes and future research needs. Biotech Adv 33: 745–755. 10.1016/j.biotechadv.2015.05.003 26008965

[mbt212840-bib-0007] Cheng, K.Y. , Lai, K.M. , and Wong, J.W.C. (2008) Effect of pig manure compost and non‐ionic surfactant Tween 80 on phenanthrene and pyrene removal from soil vegetated with *Agropyron elongatum* . Chemosphere 73: 791–797. 18672265 10.1016/j.chemosphere.2008.06.005

[mbt212840-bib-0008] Dillewijn, P.V. , Caballero, A. , Paz, J.A. , Gonzalez‐Perez, M.M. , Oliva, J.M. , and Ramos, J.L. (2007) Bioremediation of 2,4,6‐trinitrotoulene under field conditions. Environ Sci Technol 41: 1378–1383. 17593745 10.1021/es062165z

[mbt212840-bib-0009] Doty, S.L. (2008) Enhancing phytoremediation through the use of transgenics and endophytes. New Phytol 179: 318–333. 19086174 10.1111/j.1469-8137.2008.02446.x

[mbt212840-bib-0100] EEA (2000). Management of contaminated sites in Western Europe, Topic report No 13/1999. European Environment Agency: Copenhagen, Denmark.

[mbt212840-bib-0010] Enell, A. , Andersson‐Skold, Y. , Vestin, J. , and Wagelmans, M. (2016) Risk management and regeneration of brownfields using bioenergy crops. J Soils Sedim 16: 987–1000.

[mbt212840-bib-0011] Evans, P.J. , Lo, I. , Moore, A.E. , Weaver, W.J. , Grove, W.F. , and Amini, H. (2008) Rapid full‐scale bioremediation of perchlorate in soil at a large brownfield site. Remediat (Spring): 9–25.

[mbt212840-bib-0012] Friesl‐Hanl, W. , Platzer, K. , Horak, O. , and Gerzabek, M.H. (2009) Immobilising of Cd, Pb and Zn contaminated arable soils close to a former Pb/Zn smelter: a field study in Austria over 5 years. Environ Geochem Health 31: 581–594. 19283493 10.1007/s10653-009-9256-3

[mbt212840-bib-0013] Gan, V.S. , and Ng, H.K. (2012) Current status and prospects of Fenton oxidation for the decontamination of persistent organic pollutants (POPs) in soils. Chem Eng J 213: 295–317.

[mbt212840-bib-0014] Glass, D. (1999) US and International Markets for Phytoremediation, 1999–2000. Needham, MA: D. Glass Associates.

[mbt212840-bib-0101] Gerhardt, K.E. , Huang, X.D. , Glick, B.R. , and Greenberg, B.M. (2009). Phytoremediation and rhizoremediation of organic soil contaminants: potential and challenges. Plant Sci, 176, 20–30.

[mbt212840-bib-0015] Huesemann, M.H. , Hausmann, T.S. , Fortman, T.J. , Thom, R.M. , and Cullinan, V. (2009) In situ phytoremediation of PAH‐ and PCB‐ contaminated marine sediments with eelgrass (*Zostera marina* . Ecol Eng 35: 1395–1404.

[mbt212840-bib-0016] Jones, S. , Bardos, R.P. , Kidd, P.S. , Mench, M. , deLeij, F. , Hutchings, A. , *et al* (2016) Biochar and compost amendments enhance copper immobilisation and support plant growth in a contaminated soil. J Environ Manage 171: 101–112. 26850677 10.1016/j.jenvman.2016.01.024

[mbt212840-bib-0017] Kao, C.M. , and Wu, M.J. (2000) Enhanced TCDD degradation by Fenton's reagent peroxidation. J Hazard Mater 74: 197–211. 10794914 10.1016/s0304-3894(00)00161-8

[mbt212840-bib-0018] Kobayashi, T. , Murai, Y. , Tatsumi, K. , and Iimura, Y. (2009) Biodegradation of polycyclic aromatic hydrocarbons by *Sphingomonas* sp. enhanced by water‐extractable organic matter from manure compost. Sci Tot Environ 407: 5805–5810. 10.1016/j.scitotenv.2009.06.041 19660784

[mbt212840-bib-0019] Kumar, R. , Pandey, S. , and Pandey, A. (2006) Plant roots and carbon sequestration. Curr Sci 91: 885–890.

[mbt212840-bib-0020] Kuppusamy, S. , Palanisami, T. , Megharaj, M. , Venkateswarlu, K. , and Naidu, R. (2016a) Ex situ remediation technologies for environmental pollutants: a critical perspective. Rev Environ Contam Toxicol 236: 117–192. 26423074 10.1007/978-3-319-20013-2_2

[mbt212840-bib-0021] Kuppusamy, S. , Palanisami, T. , Megharaj, M. , Venkateswarlu, K. , and Naidu, R. (2016b) In situ remediation approaches for the management of contaminated sites: a comprehensive overview. Rev Environ Contam Toxicol 236: 1–115. 26423073 10.1007/978-3-319-20013-2_1

[mbt212840-bib-0022] Leigh, M.B. , Fletcher, J.S. , Fu, X. , and Schmitz, F.J. (2002) Root turnover: an important source of microbial substrates in rhizosphere remediation of recalcitrant contaminants. Environ Sci Technol 36: 1579–1583. 11999069 10.1021/es015702i

[mbt212840-bib-0023] Leigh, M.B. , Prouzova, P. , Mackova, M. , Macek, T. , Nagle, D.P. , and Fletcher, J.S. (2006) Polychlorinated biphenyl (PCB)‐degrading bacteria associated with trees in a PCB contaminated site. Appl Environ Microbiol 72: 2331–2342. 16597927 10.1128/AEM.72.4.2331-2342.2006 PMC1449058

[mbt212840-bib-0024] Mattei, P. , Cincinelli, A. , Martellini, T. , Natalini, R. , Pascale, E. , and Renella, G. (2016) Reclamation of river dredged sediments polluted by PAHs by co‐composting with green waste. Sci Tot Environ 566–567: 567–574. 10.1016/j.scitotenv.2016.05.140 27236622

[mbt212840-bib-0025] Megharaj, M. , Ramakrishnan, B. , Venkateswarlu, K. , Sethunathan, N. , and Naidu, R. (2011) Bioremediation approaches for organic pollutants: a critical perspective. Environ Int 37: 1362–1375. 21722961 10.1016/j.envint.2011.06.003

[mbt212840-bib-0026] Mench, M. , Lepp, N. , Bert, V. , Schwitzuebel, J.P. , Gawronski, S.W. , Schroder, P. , and Vangronsveld, J. (2010) Successes and limitations of phytotechnologies at field scale: outcomes, assessments and outlook from COST Action 859. J Soil Sediments 10: 1039–1070.

[mbt212840-bib-0027] Oliver, L. , Ferber, U. , Grimski, D. , Millar, K. , and Nathanail, P. (2005) The scale and nature of European Brownfields. URL http://www.cabernet.org.uk/resourcefs/417.pdf.

[mbt212840-bib-0028] Palmroth, M.R.T. , Langwaldt, J.H. , Aunola, T.A. , Goi, A. , Munster, U. , Puhakka, J.A. , and Tuhkanen, T.A. (2006) Effect of modified Fenton's reaction on microbial activity and removal of PAHs in creosote oil contaminated soil. Biodegradation 17: 131–141. 16456613 10.1007/s10532-005-6060-3

[mbt212840-bib-0029] Pelaez, A.I. , Lores, I. , Sotres, A. , Mendez‐Garcia, C. , Fernandez‐Velarde, C. , Santos, J.A. , *et al* (2013) Design and field‐scale implementation of an on site bioremediation treatment in PAH‐polluted soil. Environ Pollut 181: 190–199. 23867700 10.1016/j.envpol.2013.06.004

[mbt212840-bib-0030] Saez, J.M. , Alvarez, A. , Benemelli, C.S. , and Amorosso, M.J. (2014) Enhanced lindane removal from soil slurry by immobilised *Streptomyces* consortium. Int Biodetior Biodeg 93: 63–69.

[mbt212840-bib-0031] Semple, K.T. , Reid, B.J. , and Fermor, T.R. (2001) Impact of composting strategies on the treatment of soils contaminated with organic pollutants. Environ Pollut 112: 269–283. 11234545 10.1016/s0269-7491(00)00099-3

[mbt212840-bib-0032] Siciliano, S.D. , Germida, J.J. , Banks, K. , and Greer, C.W. (2003) Changes in microbial community composition and function during a polyaromatic hydrocarbon phytoremediation field trial. Appl Environ Microbial 69: 483–489. 10.1128/AEM.69.1.483-489.2003 PMC152433 12514031

[mbt212840-bib-0033] Tandy, S. , Healey, J.R. , Nason, M.A. , Williamson, J.C. , and Jones, D.L. (2009) Remediation of metal polluted mine soil with compost: co‐composting verses incorporation. Environ Pollut 157: 690–697. 18819736 10.1016/j.envpol.2008.08.006

[mbt212840-bib-0034] Tsao, D.T. (2003) Overview of phytotechnologies. Adv Biochem Eng Biotechnol 78: 1–50. 12674397 10.1007/3-540-45991-x_1

[mbt212840-bib-0035] UBA (2015) Register of Contaminated Sites in Germany – federal Survey, Umweltbundesant: based on data provided by all federal states; reference date 24.08.2015. URL http://www.unweltbundesamt.de/daten/bodenbelastung‐landoekosysteme/altlasten‐ihre‐sanierung.

[mbt212840-bib-0036] U.S. Environmental Protection agency (USEPA) (2002) Small business liability Relief and Brownfields Revitalization Act, Public Law 107‐118 (H.R. 2869), January 11, 2002.

[mbt212840-bib-0037] U.S. Environmental Protection agency (USEPA) (2005) Perchlorate treatment technology update. Federal facilities forum issue paper. EPA 542‐R‐05‐015.

[mbt212840-bib-0038] Van‐Camp, L. , Bujarrabal, B. , Gentile, A.R. , Jones, R.J.A. , Montanarella, L. , Olazabal, C. , and Selvaradjou, S.K. (2004) Reports of the technical working groups established under the thematic strategy for soil protection, EUR 21319 EN/4, p 872. Office of Official Publications of the European Communities, Luxembourg.

[mbt212840-bib-0039] Vangronsveld, J. , Herzig, R. , Weyens, N. , Boulet, J. , Adriaensen, K. , Ruttens, A. , *et al* (2009) Phytoremediation of contaminated soils and groundwater: lessons from the field. Environ Sci Pollut Res Int 16: 765–794. 19557448 10.1007/s11356-009-0213-6

[mbt212840-bib-0040] Waller, A.S. , Cox, E.E. , and Edwards, E.A. (2004) Percholorate‐reducing microorganisms isolated from contaminated sites. Environ Microbiol 6: 517–527. 15049925 10.1111/j.1462-2920.2004.00598.x

[mbt212840-bib-0041] Wang, L. , Chi, X.Q. , Zhang, J.J. , Sun, D.L. , and Zhou, N.Y. (2014) Bioaugmentation of a methyl parathion contaminated soil with *Pseudomonas* sp. strain WBC‐3. Int Biodetior Biodeg 87: 116–121.

[mbt212840-bib-0042] Wu, G. , Kechavarzi, C. , Li, X. , Sui, H. , Pollard, S.J. , and Coulon, F. (2013) Influence of mature compost amendment on total and bioavailable polycyclic aromatic hydrocarbons in contaminated soils. Chemosphere 90: 2240–2246. 23141842 10.1016/j.chemosphere.2012.10.003

[mbt212840-bib-0043] Zeng, G.M. , Yu, Z. , Chen, Y.N. , Zhang, J.C. , Li, H. , Yu, M. , *et al* (2011) Response of compost maturity and microbial community composition to pentachlorophenol (PCP)‐contaminated soil during composting. Biores Technol 102: 5905–5911. 10.1016/j.biortech.2011.02.088 21414773

